# Habitat-Mediated Variation in the Importance of Ecosystem Engineers for Secondary Cavity Nesters in a Nest Web

**DOI:** 10.1371/journal.pone.0090071

**Published:** 2014-02-28

**Authors:** Hugo Robles, Kathy Martin

**Affiliations:** 1 Department of Forest and Conservation Sciences, Centre for Applied Conservation Research, University of British Columbia, Vancouver, British Columbia, Canada; 2 Pacific Wildlife Research Centre, Environment Canada, Delta, British Columbia, Canada; University of Manitoba, Canada

## Abstract

Through physical state changes in biotic or abiotic materials, ecosystem engineers modulate resource availability to other organisms and are major drivers of evolutionary and ecological dynamics. Understanding whether and how ecosystem engineers are interchangeable for resource users in different habitats is a largely neglected topic in ecosystem engineering research that can improve our understanding of the structure of communities. We addressed this issue in a cavity-nest web (1999–2011). In aspen groves, the presence of mountain bluebird (*Sialia currucoides*) and tree swallow (*Tachycineta bicolour*) nests was positively related to the density of cavities supplied by northern flickers (*Colaptes auratus*), which provided the most abundant cavities (1.61 cavities/ha). Flickers in aspen groves provided numerous nesting cavities to bluebirds (66%) and swallows (46%), despite previous research showing that flicker cavities are avoided by swallows. In continuous mixed forests, however, the presence of nesting swallows was mainly related to cavity density of red-naped sapsuckers (*Sphyrapicus nuchalis*), which provided the most abundant cavities (0.52 cavities/ha), and to cavity density of hairy woodpeckers (*Picoides villosus*), which provided few (0.14 cavities/ha) but high-quality cavities. Overall, sapsuckers and hairy woodpeckers provided 86% of nesting cavities to swallows in continuous forests. In contrast, the presence of nesting bluebirds in continuous forests was associated with the density of cavities supplied by all the ecosystem engineers. These results suggest that (i) habitat type may mediate the associations between ecosystem engineers and resource users, and (ii) different ecosystem engineers may be interchangeable for resource users depending on the quantity and quality of resources that each engineer supplies in each habitat type. We, therefore, urge the incorporation of the variation in the quantity and quality of resources provided by ecosystem engineers across habitats into models that assess community dynamics to improve our understanding of the importance of ecosystem engineers in shaping ecological communities.

## Introduction

Ecosystem engineers are organisms that modulate resource availability to other organisms by maintaining or creating new habitat through physical state changes in biotic or abiotic components of the ecosystems [1]. Through this environmental modification, ecosystem engineers change the selective pressures to which other organisms are exposed (i.e. the “niche construction” process, see [2], [3]). Consequently, ecosystem engineers are thought to be major drivers of evolutionary and ecological dynamics [3], [4]. Given that niche construction via ecosystem engineering is a widespread phenomenon that structures ecological communities [3], [5], a proper knowledge of the mechanisms underlying the associations between ecosystem engineers and resource users can improve our understanding of the structure of numerous ecological communities [6].

Several recent studies show the importance of ecosystem engineers in structuring ecological populations or communities in a wide variety of environments [7]–[16]. However, the variation in the influence of particular species of ecosystem engineers in different habitat types is poorly known, despite the recognized assumption that ecosystem engineering is a context-dependent process [1], [5]. Because different habitats support communities that differ in the abundances of specific ecosystem engineers, the importance of each ecosystem engineer species for resource users may vary across habitats. Consequently, habitat may mediate the specific associations between ecosystem engineers and resource users. Some studies on burrowing mammals have found that the effects of ecosystem engineering vary spatially in extensive geographic ranges [12], [17], but whether habitat types mediate the specific associations between ecosystem engineers and resource users after controlling for the confounding effect of the large spatial variation in ecosystem engineering remains unclear. Assessment of the specific associations between ecosystem engineers and resource users in different habitat types at local spatial scales is, however, important to shed light on whether such associations vary with habitat.

Different species of ecosystem engineer may provide resources of differing quality for a particular resource user [6], [9]. Thus, ecosystem engineers can be considered as “suboptimal” or “preferred”. A high abundance of suboptimal ecosystem engineers in habitats where the preferred ecosystem engineers are scarce may allow populations of resource users to persist. However, the influence of alternative species of ecosystem engineers on local populations of resource users in different habitats has received little investigation because most previous studies have assessed the influence of only single species of ecosystem engineer in one habitat type (but see [9]). A proper assessment of how different species of ecosystem engineers can supply resource users with alternative resources in different habitats can improve our understanding of how communities subjected to ecosystem engineering processes are structured.

Cavity-nesting birds depend on tree-cavities (i.e. the critical resource) for nesting in forest ecosystems [18], [19] and are hierarchically structured in nest web communities according to their mode of acquiring cavities (e.g. [20], [21]). While primary cavity nesters are able to build their own breeding cavities through excavation (e.g. [20]), secondary cavity-nesting birds require cavities provided by avian excavators or by fungal/insect decay (e.g. [20], [22]). Fungal and insect infections induce cavity formation either directly by progressive heartwood decay or indirectly by providing suitable substrate for woodpecker excavation [23], [24]. Therefore, avian excavators and rot fungi/insects function as key ecosystem engineers that facilitate the production of nest-sites for secondary cavity nesters [1].

In this study, we aimed (i) to assess whether habitat mediates the specific associations between ecosystem engineers and resource users in a cavity nest-web community, and (ii) to examine whether different species of ecosystem engineers provide resource users with alternative resources in different habitats. To assess the variation in the importance of the ecosystem engineers in relation to habitat, we investigated the relationships between the presence of nests of two secondary cavity nesters (the mountain bluebird *Sialia currucoides* and the tree swallow *Tachycineta bicolor*) in two different habitats (aspen groves and continuous mixed deciduous-coniferous forests), and the density of cavities supplied by four types of ecosystem engineers: three cavity excavating birds (northern flicker *Colaptes auratus*, red-naped sapsucker *Sphyrapicus nuchalis*, hairy woodpecker *Picoides villosus*) and rot fungi/insects.

Bluebirds are more likely to nest in cavities supplied by northern flickers, hairy woodpeckers and rot-fungi/insects than in cavities supplied by red-naped sapsuckers [6]. Swallows, however, are more likely to nest and to successfully produce fledglings in hairy woodpecker and sapsucker cavities than in flicker cavities [6]. We expected a higher density of flicker cavities relative to other cavity types in aspen groves, as flickers reach very high abundances in these patchy forests surrounded by extensive grasslands suitable for foraging [25]. We predicted that the high abundance of flicker cavities in aspen groves would lead to a high use of these cavities by the secondary cavity nesters (i.e. the resource users). This can be true particularly for bluebirds, but not so much for swallows, which avoid flicker cavities (see above). In continuous forests, however, we expected less difference in the density of cavities supplied by different ecosystem engineers compared to aspen groves. Consequently, we predicted a more similar contribution of the density of cavities provided by each ecosystem engineer on the presence of secondary cavity nesters in continuous forests.

## Methods

### Ethics statement

The surveys were conducted primarily on public lands. The Department of National Defence allowed us to conduct field research on two sites and lease holders allowed us access to three sites. All surveys were based on non-invasive observational procedures to minimize disturbance on birds. The research was approved by the Animal Care & Biosafety Committee of the University of British Columbia (permit numbers: A04-0101 to A07-130). All field activities were in agreement with federal and provincial legislation.

### Study area and species

We collected the data in 35 sites (7–32 ha) within 50 km of Williams Lake (51° 52′ N, 122° 21′ W) in interior British Columbia, Canada. The study included two forest types: continuous mixed forests (27 sites) and aspen groves (8 sites). Continuous mixed deciduous-coniferous forest sites (≥80 years) were composed mainly of Lodgepole pine (*Pinus contorta* var. *latifolia*, 42% of trees), Douglas-fir (*Pseudotsuga menziesii*, 28%), hybrid white spruce (*Picea glauca x engelmannii*, 18%) and trembling aspen (*Populus tremuloides*, 12%), whereas black cottonwood (*Populus balsamifera*), alder (*Alnus* spp.), paper birch (*Betula papyrifera*), and willow (*Salix* spp.) were present in very low proportions [26]. Continuous mixed forests were occasionally disrupted by small grasslands, shallow ponds and selective harvesting. Aspen grove sites consisted of a few small clusters of trees (0.2–5 ha) surrounded by an extensive matrix of grasslands and shallow ponds. Aspen groves were composed principally of trembling aspen (54%) and, to a lesser extent, of lodgepole pine (38%) and Douglas-fir (8%) [26].

Mountain bluebirds and tree swallows are secondary cavity-nesting passerine birds. Because both insectivores require open areas for foraging [27], [28], the extensive matrix of grasslands and shallow ponds surrounding aspen groves provides swallows and bluebirds with large areas of suitable foraging habitat. The availability of foraging habitat is more limited in continuous forests, where these birds forage over the interspersed grasslands, shallow ponds and clear-cuts within the forest. While populations of mountain bluebirds remain stable in North America [27], tree swallow populations have declined in recent decades in the areas of greatest swallow density within its range [28], [29]. Populations of both secondary cavity nesters increased on our sites during the course of this study [30].

### Field methods

We searched for nests of cavity excavators and secondary cavity nesters in May–July, 1995–2011 (average of 10.8±0.5 SE years of data per site). During nest surveys, which were conducted for an average of 6–7 observer-hours per sampling site per week, nest cavities were found by observing behavior of adults and listening for begging nestlings. We found most nests during the laying or early incubation stage. To inspect cavity contents, we used either a ladder, flashlights and mirrors, or a video camera system mounted in a pole (TreeTop Peeper; Sandpiper Technologies, Manteca, CA). We considered cavities with at least one egg or nestling to contain active nests.

Starting in 1999, we systematically monitored the cavities we found during routine searches in previous years until they were no longer available for nesting (details in [26]), which allowed us to calculate the density of cavities supplied by ecosystem engineers in a given site and year [6]. We excluded data from 1995 to 1998 from calculations of cavity densities because the survey effort differed from that of the later study years and the cavity formation agent (ecosystem engineer) of some cavities at the beginning of the study was unknown. We, therefore, calculated cavity density supplied by ecosystem engineers from 1999 to 2011 during which we knew the cavity history and the identity of the ecosystem engineer that formed the cavity [6].

### Statistical analyses

#### Density of cavities supplied by ecosystem engineers

We used linear mixed-effects models (LMEs) to examine potential differences in the density of cavities (cavities ha^−1^) supplied by ecosystem engineers between continuous forests and aspen groves. The response variable was the density of cavities supplied by the ecosystem engineers for each site-year, whereas habitat type (continuous forests vs. aspen groves) was fitted as a categorical fixed term. Site identity was fitted as a random term to control for multiple observations within the same sites across years.

We also assessed whether particular species of ecosystem engineers supplied higher densities of cavities than others in continuous forests and in aspen groves. For each habitat type, we performed a LME with the density of cavities as the response variable, the identity of each ecosystem engineer as a fixed term and site identity as a random term. All cavity densities in this paper were logarithmic-transformed. LMEs were conducted using the package “nlme” [31] in R 2.14.1 [32].

#### Presence of secondary cavity nesters in relation to the density of cavities supplied by ecosystem engineers

We used generalized linear mixed models (GLMMs) with binomial error distributions and logit link functions to assess the relationships between the presence of nesting bluebirds or swallows (presence of active nests in a given site and year  = 1, no active nests  = 0) and the density of cavities (cavities ha^−1^) supplied by the ecosystem engineers in continuous mixed forests and aspen groves. Site identity was fitted as a random term. In addition, site size (ha) was fitted as a fixed term to control for its potential effect on the detection of nests. GLMMs were conducted using the package “lme4” [33] in R 2.14.1 [32].

We ran one set of models for each species of secondary cavity nester and each habitat type. Each model set was composed of 7 models. The first 4 models comprised the density of cavities supplied by each ecosystem engineer and site size as fixed terms. Another model included, in addition to site size, the density of cavities of the ecosystem engineers preferred by bluebirds (flickers, hairy woodpeckers and rot-fungi/insects; see above) or swallows (hairy woodpeckers and sapsuckers) for nesting. One model was composed of site size only (i.e. a null model). Finally, one model included site size and the density of cavities supplied by all the ecosystem engineers. This modeling approach allowed us to examine the importance of the ecosystem engineers by comparing models with the density of cavities supplied by each engineer species with models that included the preferred cavities for each secondary cavity nester as well as with null models and with models that included all cavities in the sites.

We used an information-theoretic approach [34] to assess the importance of the density of cavities facilitated by the ecosystem engineers on the presence of nesting swallows and bluebirds in a given site and year. We ranked the models according to Akaike's Information Criterion corrected for small sample sizes (AICc) and Akaike model weights [34], [35]. Models with low AICc values are better supported by the data. Akaike model weights quantify the support of every model by the data, where higher weights indicate better explanatory power and the sum of all model weights is 1 [34].

## Results

### Density of cavities supplied by ecosystem engineers

**Figure 1:**
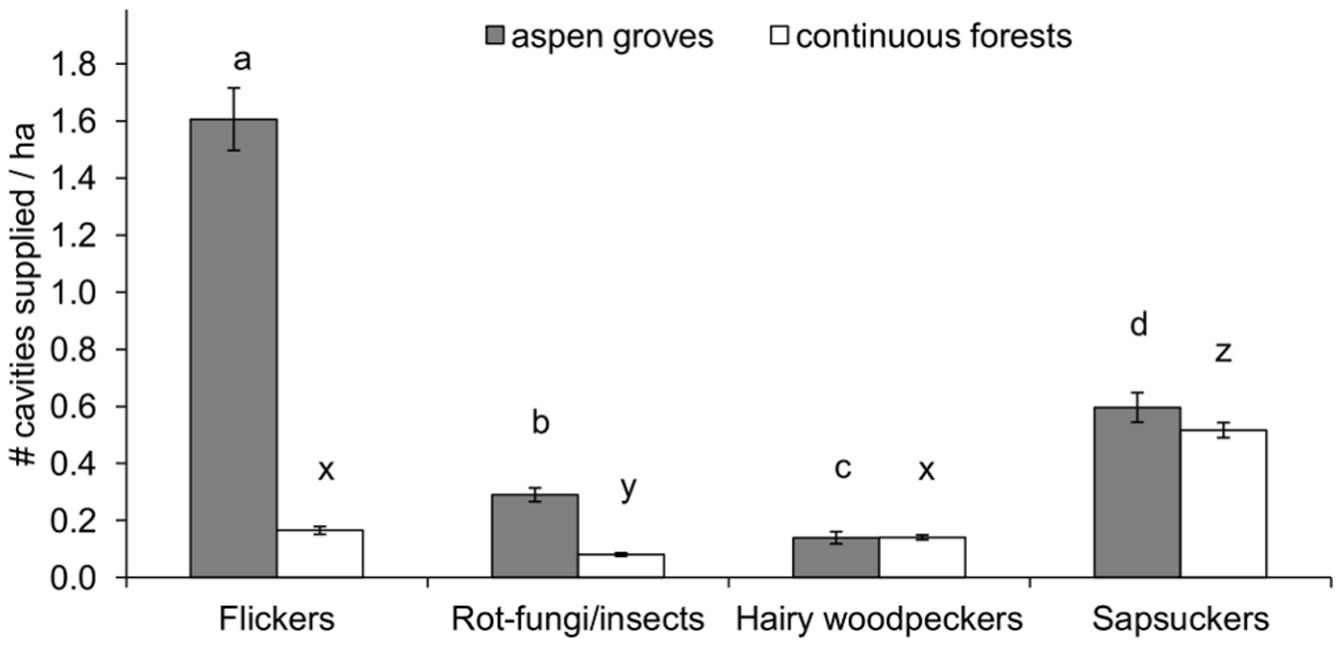
Average density of cavities supplied by the ecosystem engineers. Different letters above standard error bars indicate significant differences in cavity densities supplied by the ecosystem engineers for each habitat type (aspen grove or continuous forest; Tukey's post-hoc test for the models in table 1: all *p*<0.001).

Aspen groves had higher densities of cavities supplied by flickers (parameter estimate ± SE  = −2.617±0.372, *t* = −7.027, *p*<0.001) and rot-fungi/insects (parameter estimate ± SE  = −1.347±0.263, *t* = −5.122, *p*<0.001) than continuous forests (Fig. 1). In contrast, the densities of sapsucker (parameter estimate ± SE  = −0.503±0.500, *t* = −1.006, *p* = 0.324) and hairy woodpecker (parameter estimate ± SE  = −0.179±0.453, *t* = −0.396, *p* = 0.697) cavities did not differ significantly between both forest types (Fig. 1).

In aspen groves, flickers supplied the highest density of cavities, followed by sapsuckers, rot-fungi/insects and hairy woodpeckers (Table 1, Fig. 1). In continuous forests, sapsuckers provided significantly higher densities of cavities than all the other ecosystem engineers, and the densities of cavities supplied by hairy woodpeckers and flickers were significantly higher than those supplied by rot-fungi/insects (Table 1, Fig. 1).

**Table 1:** Parameter estimates of models that examined the differences in the density of cavities supplied by the ecosystem engineers in aspen groves and in continuous forest sites.

Habitat	Parameters	Estimate ± SE	*t*	*p*
Aspen grove	Intercept	−2.507±0.359	−6.984	<0.001
	Ecosystem engineer (Flickers)	2.853±0.109	26.227	<0.001
	Ecosystem engineer (Rot-fungi/insects)	1.139±0.086	10.363	<0.001
	Ecosystem engineer (Sapsuckers)	1.839±0.109	16.904	<0.001
Continuous forest	Intercept	−2.194±0.108	−20.389	<0.001
	Ecosystem engineer (Flickers)	−0.047±0.081	−0.579	0.563
	Ecosystem engineer (Rot-fungi/insects)	−0.542±0.104	−5.195	<0.001
	Ecosystem engineer (Sapsuckers)	1.160±0.073	15.916	<0.001

### Nesting cavities of secondary cavity nesters

#### Nesting cavities of bluebirds

**Figure 2:**
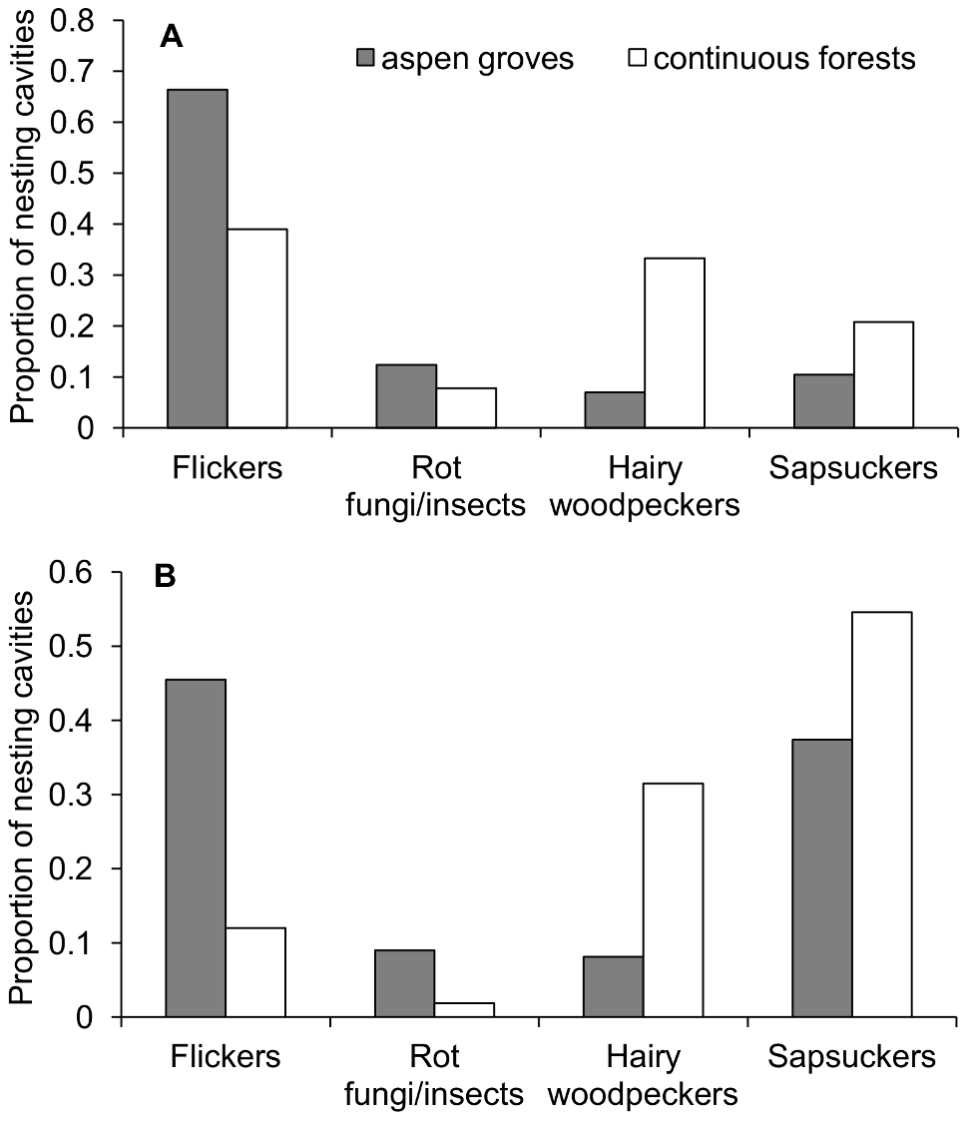
Proportion of nesting cavities supplied by the ecosystem engineers. The proportion of nests of bluebirds (A) and swallows (B) in cavities supplied by each ecosystem engineer are shown for aspen groves and continuous forests sites. Sample sizes: 229 and 77 nesting cavities of bluebirds in aspen groves and continuous forests, respectively; 222 and 108 nesting cavities of swallows.

In aspen groves, flickers supplied most nesting cavities to bluebirds, followed by rot-fungi/insects, sapsuckers and hairy woodpeckers (Fig. 2A). In continuous forests, bluebirds nested in flicker and hairy woodpecker cavities in similar proportions (Fig. 2A). Sapsucker cavities were used in lower proportions than flicker and hairy woodpecker cavities in continuous forests, whereas only a few cavities supplied by rot-fungi/insects were used by bluebirds for nesting (Fig. 2A).

#### Nesting cavities of swallows

In aspen groves, swallows mostly nested in flicker cavities and, to a lesser extent, in sapsucker cavities (Fig. 2B). Hairy woodpeckers and rot-fungi/insects provided few nesting cavities to swallows in aspen groves (Fig. 2B). In continuous forests, swallows mostly nested in sapsucker and hairy woodpecker cavities, whereas flickers and rot-fungi/insects provided swallows with few nesting cavities (Fig. 2B).

### Presence of secondary cavity nesters in relation to the density of cavities supplied by ecosystem engineers

#### Presence of bluebird nests

**Figure 3:**
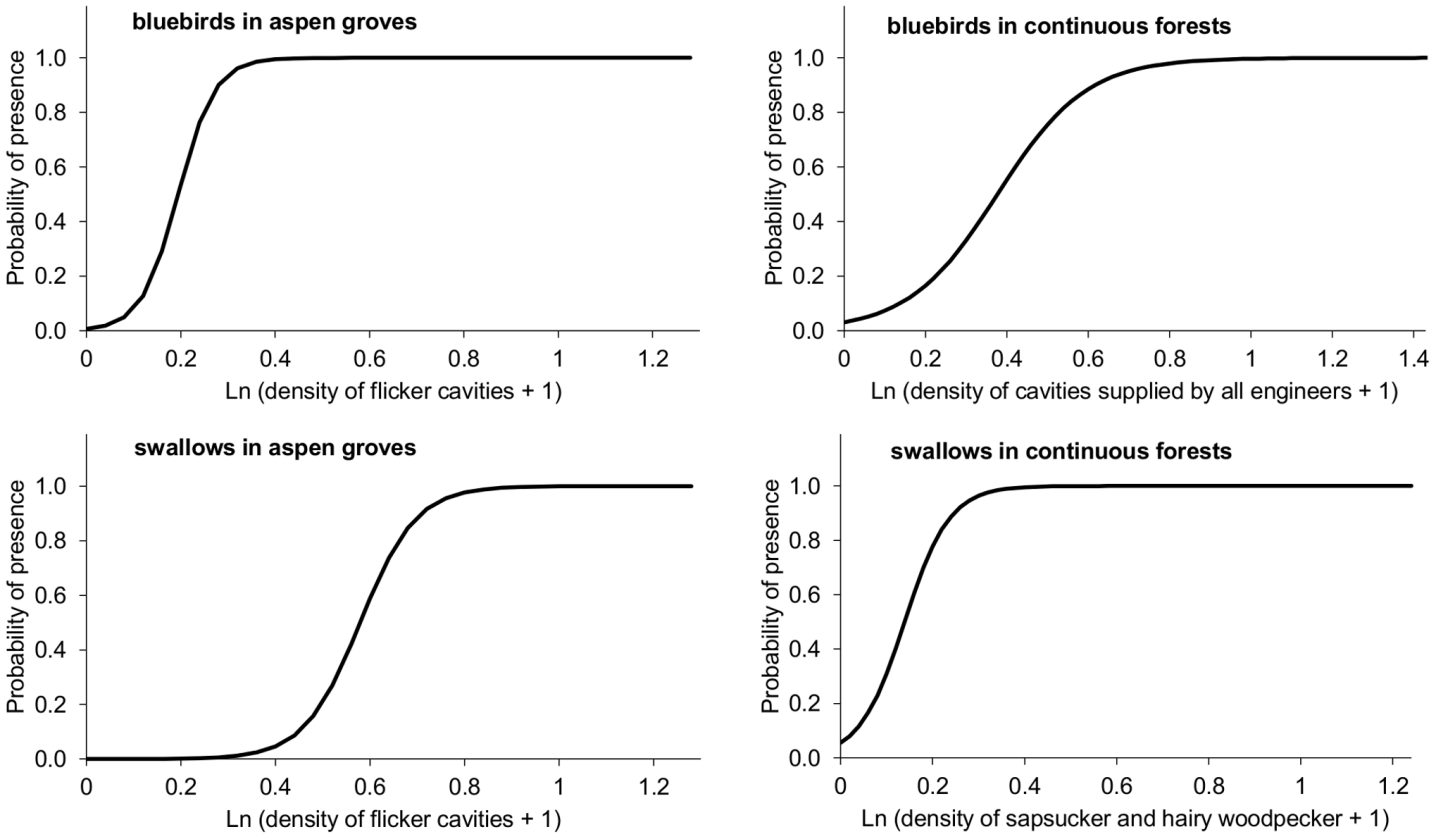
Probability of presence of bluebird and swallow nests in relation to cavity density supplied by the ecosystem engineers. The probability of presence was defined as the probability that a given aspen grove or continuous forest site had at least one cavity used by bluebirds or swallows for nesting in a given year. Presence probabilities are the predicted probabilities calculated from the best models that depicted the presence of nests in Table 2.

In aspen groves, we found bluebird nests in 33 of 39 site-years (84.6%). Model selection of analyses that examined the presence of bluebird nests in aspen groves yielded two high ranked models that accounted for most weight in the candidate model set (Table 2). The best model included the density of flicker cavities as a predictor of the presence of bluebird nests, whereas the second-ranked model included the density of the preferred cavities (i.e. supplied by flickers, hairy woodpeckers and rot-fungi/insects). The presence of nesting bluebirds was positively related to the density of flicker cavities (parameter estimate ± SE  = 25.8±12.6 *z* = 2.053, *p* = 0.040, Fig. 3) and to the density of the preferred cavities (parameter estimate ± SE  = 16.6±7.3, *z* = 2.277, *p* = 0.023). However, among the preferred cavities, the models that included the density of cavities supplied by hairy woodpeckers and rot-fungi/insects had low model weights and AICc values similar to the null model (ΔAICc<3, see Table 2), which indicates low influence of these cavities on the presence of bluebird nests.

**Table 2:** Model selection of analyses that examined the presence of bluebird or swallow nests in aspen groves and continuous forest sites.

Species	Habitat	Models	df	LogLik	AICc	ΔAICc	Weight
Bluebird	Aspen grove	Flickers	4	−6.03	21.24	0.00	0.67
		Flickers + hairy woodpeckers + rot-fungi/insects	4	−6.88	22.94	1.70	0.29
		All ecosystem engineers	4	−8.90	26.99	5.75	0.04
		Hairy woodpeckers	4	−12.09	33.36	12.12	0.00
		Null model	3	−14.70	36.09	14.85	0.00
		Sapsuckers	4	−13.73	36.64	15.40	0.00
		Rot-fungi/insects	4	−13.81	36.79	15.55	0.00
Bluebird	Continuous forest	All ecosystem engineers	4	−107.26	222.67	0.00	0.97
		Sapsuckers	4	−111.09	230.33	7.66	0.02
		Flickers + hairy woodpeckers + rot-fungi/insects	4	−112.43	233.01	10.34	0.01
		Flickers	4	−119.55	247.26	24.58	0.00
		Hairy woodpeckers	4	−119.72	247.59	24.91	0.00
		Null model	3	−129.24	264.56	41.89	0.00
		Rot-fungi/insects	4	−128.47	265.08	42.41	0.00
Swallow	Aspen grove	Flickers	4	−5.62	20.42	0.00	0.58
		All ecosystem engineers	4	−6.11	21.40	0.98	0.36
		Sapsuckers + hairy woodpeckers	4	−8.68	26.53	6.11	0.03
		Sapsuckers	4	−8.75	26.68	6.26	0.03
		Null model	3	−12.25	31.18	10.76	0.00
		Rot-fungi/insects	4	−11.02	31.21	10.79	0.00
		Hairy woodpeckers	4	−11.87	32.91	12.50	0.00
Swallow	Continuous forest	Sapsuckers + hairy woodpeckers	4	−150.78	309.70	0.00	0.46
		All ecosystem engineers	4	−150.82	309.80	0.10	0.44
		Sapsuckers	4	−152.25	312.65	2.95	0.10
		Hairy woodpeckers	4	−156.05	320.24	10.54	0.00
		Flickers	4	−160.26	328.66	18.96	0.00
		Null model	3	−164.22	334.53	24.82	0.00
		Rot-fungi/insects	4	−164.18	336.51	26.80	0.00

N = 39 and 276 site-years in aspen groves and continuous forests, respectively. The presence of nests was related to the density of cavities (logarithmic-transformed) supplied by the ecosystem engineers: northern flickers, red-naped sapsuckers, hairy woodpeckers and rot-fungi insects. Site size was fitted as a fixed term in all models to control for its potential effect on nest detection. LogLik: log-likelihood, AICc: AIC corrected for small sample size, ΔAICc: difference in AICc to the best model. Models of each model set for each bird species and habitat type are ranked according to their Akaike weight (Weight).

In continuous forests, we found bluebird nests in 64 of 276 site-years (23.2%). Model selection of analyses that examined the presence of bluebird nests in continuous forests yielded a single high ranked model that accounted for most weight in the candidate model set (Table 2). The best model included the density of cavities supplied by all the ecosystem engineers. The presence of bluebird nests was most strongly and positively associated with the density of cavities supplied by all the ecosystem engineers (parameter estimate ± SE  = 6.020±1.066, *z* = 5.649, *p*<0.001, Fig. 3).

#### Presence of swallow nests

In aspen groves, we found swallow nests in 32 of 39 site-years (82.1%). Model selection of analyses that examined the presence of swallow nests in aspen groves yielded two high ranked models that accounted for most weight in the candidate model set (Table 2). Similar to bluebird analyses, the best model included the density of flicker cavities as a predictor of swallow nest presence. The presence of nesting swallows in a given aspen grove and year was positively related to the density of flicker cavities (parameter estimate ± SE  = 16.94±8.57, *z* = 1.977, *p* = 0.048, Fig. 3). The second ranked model included the density of cavities supplied by all the ecosystem engineers (parameter estimate ± SE  = 13.63±7.01, *z* = 1.950, *p* = 0.051). However, the models composed of the density of cavities supplied by sapsuckers, hairy woodpeckers or rot-fungi/insects had low model weights and AICc values similar to the null model (ΔAICc<4, see Table 2), indicating low influence of these three ecosystem engineers on swallows nesting in aspen groves.

In continuous forests, we found swallow nests in 93 of 276 site-years (33.7%). Model selection of analyses that examined the presence of swallow nests in continuous forests yielded two high ranked models that accounted for most of the weight in the candidate model set (Table 2). The best model included the density of hairy woodpecker and sapsucker cavities as a predictor; that is, the cavities preferred by swallows for nesting. The presence of swallow nests was positively related to the density of these preferred cavities (parameter estimate ± SE  = 4.024±0.817, *z* = 4.927, *p*<0.001, Fig. 3). The second best model included the density of cavities supplied by all the ecosystem engineers (parameter estimate ± SE  = 3.671±0.736, *z* = 4.989, *p*<0.001). However, the models that included the density of cavities supplied by flickers and rot-fungi/insects had low model weights and AICc values similar to the null model (ΔAICc<5, see Table 2), which indicates low influence of these cavities on the presence of swallow nests in continuous forests.

## Discussion

While niche construction via ecosystem engineering is considered to be a widespread phenomenon that structures ecological communities, the variation in the importance of ecosystem engineers across environmental gradients (e.g., across different habitats) is poorly understood [3], [5]. Our results suggest that habitat type may mediate the inter-specific associations between ecosystem engineers and resource users. In aspen groves, the presence of bluebird and swallow nests was mainly and positively associated with the density of cavities supplied by flickers, which provided most nesting cavities to bluebirds and swallows (66% and 45% of nesting cavities, respectively; Fig. 2). In continuous forests, however, the presence of bluebird nests was positively related to the density of cavities supplied by all ecosystem engineers. In addition, the presence of swallow nests in continuous forests was positively associated with the density of cavities supplied by sapsuckers and hairy woodpeckers, which provided 86% of nesting cavities used by swallows (Fig. 2).

To our knowledge, only one other study [9] has assessed the influence of habitat at local spatial scales to determine the variation in the associations between multiple ecosystem engineers and resource users in ecological communities. In their research, Machicote et al. [9] found that hairy armadillos (*Chaetophractus villosus*) produce suitable nest sites for burrowing owls (*Athene cunicularia*) in recently burned areas or areas maintained by anthropogenic disturbance, but in other habitat types plains vizcachas (*Lagostomus maximus*) play a major role as ecosystem engineers for these owls. These results suggest that habitat may mediate the associations between ecosystem engineers (i.e. vizcachas and armadillos) and resource users (i.e. owls) [9], which matches our results showing that habitat may mediate the inter-specific associations between cavity facilitators and secondary cavity users in a cavity-nest web community.

A better understanding of the context-dependent effects of ecosystem engineering can improve our knowledge of how ecological systems are structured [5]. Our results may help elucidate the mechanisms underlying the context-dependent variation in the importance of different ecosystem engineers. In our study system, both resource quantity and quality may explain the associations of resource users and ecosystem engineers in different habitats. In aspen groves, the presence of secondary cavity nesters was mainly related to the density of cavities supplied by flickers, which provided the most abundant cavities in this habitat type (Fig. 1). Flickers provide suitable nesting cavities to bluebirds, but swallows are less likely to occupy flicker cavities compared to cavities supplied by the other ecosystem engineers [6], suggesting that the high abundance (rather than the quality) of flicker cavities determines the high use of these cavities by swallows in aspen groves. Despite the relatively low abundance of sapsucker cavities compared to flicker cavities in aspen groves (Fig. 1), sapsuckers supplied 37% of the nesting cavities used by swallows (Fig. 2), possibly because these woodpeckers provide suitable cavities to swallows [6]. In continuous forests, despite the low abundance of hairy woodpecker cavities (Fig. 1), hairy woodpeckers provided approximately one third of the nesting cavities to both bluebirds and swallows (Fig. 2). The high use of hairy woodpecker cavities in continuous forests may be associated with the high quality of these cavities for both secondary cavity nesters, as cavities produced by hairy woodpeckers are the most likely to be occupied by swallows and bluebirds [6]. In addition, sapsuckers provided high numbers of nesting cavities to swallows in continuous forests (Fig. 2), perhaps because sapsuckers provided the most abundant cavities in this habitat type (Fig. 1). The small entrance size of many sapsucker cavities may denote that they are not all available for use by bluebirds [6], but even so, sapsuckers still provided 21% of the nesting cavities used by bluebirds in continuous forests. This may be due to the high cavity abundance that sapsuckers provided in this habitat type, increasing the probability that some sapsucker cavities were large enough to be accessible to bluebirds. These results suggest that the identification of the key ecosystem engineers for each resource user requires considering both the quantity and quality of the resources supplied by the ecosystem engineers in each habitat type.

Understanding how communities are structured requires a proper identification of the associations between ecosystem engineers and resource users that elucidate whether and when ecosystem engineers are functionally interchangeable [9]. The positive relationship between the presence of swallow nests and the density of flicker cavities in aspen groves suggests that nesting in suboptimal flicker cavities may be an acceptable alternative for swallows in this habitat type (flickers provided more nesting cavities to swallows in aspen groves than the other ecosystem engineers, Fig. 2). However, the exclusive use of flicker cavities might not guarantee the long-term persistence of swallow populations because swallows are less likely to produce fledglings in nesting cavities supplied by flickers than in those supplied by the other ecosystem engineers [6]. Our results also suggest that some ecosystem engineers may be functionally interchangeable for bluebirds, which used high numbers of flicker cavities in aspen groves but also high numbers of cavities supplied by hairy woodpeckers and sapsuckers in continuous forests (Fig. 2). Because the reproductive output of bluebirds nesting in cavities supplied by flickers, sapsuckers and hairy woodpeckers does not differ significantly [6], we found no evidence of fitness differences for bluebirds nesting in cavities supplied by these three different ecosystem engineers.

Cavities created by fungal or insect decay appear to be less important than those supplied by avian excavators, as avian excavators provide most nesting sites to secondary cavity nesters in North America (e.g. [21], [36]–[40]). Our results also suggest a low influence of cavities created by fungal/insect decay on secondary cavity nesters, as the density of these cavities was not strongly correlated with the presence of cavities used by bluebirds and swallows in either habitat type. Moreover, both secondary cavity nesters used few cavities created by fungal/insect decay for nesting (2–12% of nesting cavities, Fig. 2). In addition, cavities created by fungal/insect decay are less likely to be used by swallows than hairy woodpecker cavities [6], which may explain why low-abundant hairy woodpecker cavities, but not cavities created by fungi/insects, were strongly associated with the presence of nesting swallows in continuous forests.

Bluebirds and swallows were less likely to nest in continuous forests than in aspen groves. One possibility is that bluebirds and swallows are less likely to occupy continuous forest sites because these sites have lower abundance of cavities supplied by the ecosystem engineers compared to aspen groves (Fig. 1). Another possibility is that aspen groves provide bluebirds and swallows with additional habitat resources. Supporting this hypothesis, cavities in aspen groves are more likely to be used by bluebirds and swallows after controlling for the cavity formation agent (i.e. ecosystem engineer identity) than those in continuous forests [6]. The high availability of open areas (grasslands and shallow ponds) surrounding aspen groves may provide swallows and bluebirds with suitable foraging habitat [27], [28], whereas the availability of foraging habitat for these species is more limited in continuous forests. Other potential factors such as increased levels of predation or parasitism in continuous forests compared to aspen groves may also explain the lower presence of nesting bluebirds and swallows in continuous forests.

To summarize, by assessing the relationships between the presence of nests of two secondary cavity-nesting birds, and the density of cavities supplied by multiple ecosystem engineers in contrasting habitats, we show that ecosystem engineers may be interchangeable for resource users. The interchangeability of resources provided by different engineers was probably mediated by habitat type, as the two habitats supported different amounts of cavities supplied by each ecosystem engineer. Determining the generality of our results will require further evaluations of the associations between multiple ecosystem engineers and resource users in different habitats to assess the interchangeability of resources supplied by ecosystem engineers in different settings and communities. Such a study approach would also improve our understanding of the variation in ecosystem engineering effects across environmental gradients, which in turn can improve the predictions on the general effects of ecosystem engineering on abiotic and biotic processes [5], [41]. Overall, we recommend incorporating the variation in the quantity and quality of resources provided by ecosystem engineers across habitats into models that assess community structure and dynamics to provide a better understanding of the importance of ecosystem engineers in shaping ecological communities.
